# Fine Particulate Matter From 2020 California Wildfires and Mental Health–Related Emergency Department Visits

**DOI:** 10.1001/jamanetworkopen.2025.3326

**Published:** 2025-04-04

**Authors:** Youn Soo Jung, Mary M. Johnson, Marshall Burke, Sam Heft-Neal, Melissa L. Bondy, R. Sharon Chinthrajah, Mark R. Cullen, Lorene Nelson, Caleb Dresser, Kari C. Nadeau

**Affiliations:** 1 Sean N. Parker Center for Allergy and Asthma Research, Stanford University, Stanford, California; 2 Department of Environmental Health, Harvard, Boston, Massachusetts; 3 Department of Earth System Science, Stanford University, Stanford, California; 4 Center on Food Security and the Environment, Stanford University, Stanford, California; 5 Department of Epidemiology and Population Health, Stanford University, Stanford, California; 6 Stanford Center for Population Health Sciences, Stanford University, Los Altos Hills, California; 7 Harvard University T H Chan School of Public Health, Boston, Massachusetts

## Abstract

**Importance:**

A growing body of research suggests that exposure to fine particulate matter (PM_2.5_; particle size 2.5 microns or smaller) may be associated with mental health outcomes. However, the potential impact of wildfire-specific PM_2.5_ exposure on mental health remains underexplored.

**Objective:**

To investigate whether wildfire-specific PM_2.5_ exposure may be associated with emergency department (ED) visits for mental health conditions, including all-cause and for psychoactive substance use, nonmood psychotic disorders, anxiety, depression, and other mood-affective disorders during the extensive 2020 California wildfire season.

**Design, Setting, and Participants:**

This cross-sectional study used data on ED visits from July to December 2020 obtained from the California Department of Health Care Access and Information (HCAI). Eligible participants were California residents who presented to an ED in California for mental health conditions without COVID-19. The data were analyzed between July 2020 and December 2020.

**Exposure:**

Wildfire-specific PM_2.5_ exposure (with up to 7-day lags) based on participants’ residential zip codes.

**Main Outcomes and Measures:**

Daily ED visit counts for all-cause and disease-specific mental health conditions (F00-F99) identified using *International Statistical Classification of Diseases and Related Health Problems, Tenth Revision (ICD-10)* codes at zip code tabulation areas.

**Results:**

Between July and December 2020, there were 86 609 ED visits for mental health conditions (median [IQR] patient age, 38 [27-54] years; 40 272 female [46.5%]; 10 657 Black [12.3%], 30 044 Hispanic [34.7%], 35 145 White [40.6%]). Visits included psychoactive substance use (23 966 [27.6%]), nonmood psychotic disorders (16 714 [19.3%]), anxiety (26 711 [30.8%]), depression (10 422 [12.0%]), and other mood-affective disorders (5338 [6.2%]). During peak wildfire months, the median (IQR) daily concentration of wildfire-specific PM_2.5_ increased to 11.9 (3.9-32.5) μg/m^3^. A 10-μg/m^3^ increase in wildfire-specific PM_2.5_ was associated with higher ED visits for all-cause mental conditions (cumulative relative risk [cRR] over lag 0-7 days, 1.08; 95% CI, 1.03-1.12), depression (cRR over lag 0-7 days, 1.15; 95% CI, 1.02-1.30), other mood-affective disorders (cRR over lag 0-7 days, 1.29; 95% CI, 1.09-1.54), and anxiety (cRR over lag 0-4 days, 1.06; 95% CI, 1.00-1.12). Subgroup analyses suggested that wildfire smoke was associated with disproportionately increased ED visits among female individuals (eg, depression: cRR over lag 0-4 days, 1.17; 95% CI, 1.03-1.32) and young people (other mood-affective disorders: cRR over lag 0-4 days, 1.46; 95% CI, 1.08-1.98). Effect modification by race was found, with non-Hispanic Black individuals having an increased risk of ED visits for other mood-affective disorders (cRR over lag 0-5 days, 2.35; 95% CI, 1.56-3.53) and Hispanic individuals an increased risk for visits for depression (cRR over lag 0-7 days, 1.30; 95% CI, 1.06-1.59).

**Conclusions and Relevance:**

Wildfire smoke exposure was associated with significantly increased odds of subsequent ED visits for mental health conditions in this cross-sectional study, with varying lag times for different subconditions and demographic groups.

## Key Points

**Question:**

Is there an association of wildfire-specific fine particulate matter (PM_2.5_) exposure with mental health–related emergency department (ED) visits during wildfire seasons?

**Findings:**

This cross-sectional analysis of 86 668 ED visits for mental health conditions during the severe 2020 California wildfires found that exposure to wildfire-specific PM_2.5_ was associated with a significant increase in mental health–related ED visits, particularly for young children, minority racial and ethnic groups, and women.

**Meaning:**

These findings suggest a potential link between wildfire-specific PM_2.5_ exposure and mental health outcomes; health care professionals and systems should prepare for a possible increase in demand for mental health–related emergency services during wildfire events.

## Teaser

This cross-sectional study examines the association of exposure to fine particulate matter following the 2020 California wildfires with emergency department (ED) mental health–related treatment among adults.

## Introduction

The intensity, frequency, and duration of wildfires have been increasing across the US in recent years due to regional and global warming trends. This has resulted in increased loss, damage, and cost, along with deteriorating air quality from wildfire smoke containing pollutants like fine particulate matter (PM_2.5_; particle size of 2.5 microns or smaller). Previous studies have suggested the association between wildfire-specific PM_2.5_ and increased respiratory and cardiovascular hospital visits, especially among vulnerable populations.^1,2^ However, the potential effects of wildfire-specific PM_2.5_ on mental health remain less understood.^3,4^

Most wildfire studies have primarily focused on the mental consequences of traumatic experiences, such as life-threatening experiences, property loss, evacuation, and recovery-related stress.^4,5^ Several limited studies have investigated smoke exposure as a cause of mental health issues. Temporal comparison studies have found no clear link between hospital visits and wildfire smoke.^6^ While some studies have examined hospital visits and wildfire PM_2.5_ exposure, the results were unclear or showed no clear patterns.^6,7,8^ However, a 2024 paper using wildfire PM_2.5_ reported a positive association with ED visits for anxiety disorder.^9^

Although research on wildfire PM_2.5_ is still limited, evidence from the epidemiological literature on the short-term effects of ambient air pollution (AAP) suggests that exposure to high levels of PM_2.5_ may increase hospital admissions for mental health disorders, including anxiety, depression, suicide, and psychotic episodes.^10,11,12,13^ Inhaled PM_2.5_ can reach the brain, potentially causing neuroinflammation, oxidative stress, cerebral vascular damage, and neurodegenerative pathology.^14^ Existing research suggests that wildfire PM_2.5_ may pose greater mental health risk than ambient PM_2.5_ due to its complexity and toxicity.^15^

In 2020, California had the most severe wildfire season on record, with over 70% of the population enduring unhealthy air quality for over 100 days.^16^ There were 98 wildfires that burned more than 1000 acres (maximum, 1 032 699 acres), lasting a mean average of 48 days (range, 2-140 days; median, 35 days). This large-scale event exposed a diverse population to variable wildfire PM_2.5_ concentrations, allowing for more generalizable outcomes than previous studies focused on smaller or localized wildfires (eTable, eFigure 1 in Supplement 1).

In this cross-sectional study, we investigated the association of wildfire-specific PM_2.5_ with ED visits for mental health conditions—including all causes, depression, anxiety, and other mental disorders—during the 2020 California wildfire season. Our study aims to provide broadly applicable results for understanding the potential short-term impact of wildfire smoke on mental health ED visits in the general population. We also explore potentially vulnerable groups by race, ethnicity, and health insurance information, which was previously understudied.^9,10,11,12,13^ As wildfires become more frequent and severe due to climate change, these findings can contribute to developing preventive strategies to mitigate mental health risks associated with wildfire-specific PM_2.5_ exposure.

## Methods

### Study Design and Data Source

This study used data on ED visits at California-licensed hospitals obtained from Health Care Assessment and Information (HCAI). Eligible visits were between July 1 and December 31, 2020. Data included sex, self-reported race and ethnicity, resident zip code, hospital encounter information, primary diagnoses, and payer information. The study was reviewed and approved by the Stanford University institutional review board and Committee for the Protection of Human Subjects under the California Health and Human Services Agency, and was exempt from informed consent requirements because it was deidentified data. The study followed the Strengthening the Reporting of Observational Studies in Epidemiology (STROBE) reporting guidelines for observational studies.

### Outcome of Interest

ED encounter diagnoses were identified using the *International Statistical Classification of Diseases and Related Health Problems, Tenth Revision (ICD-10)* codes for mental health conditions, including all-cause mental disorders, psychoactive substance use disorders, nonmood psychotic disorders, other mood-affective disorders, depression, and anxiety (Table 1). Only patients with a primary ED diagnosis code for mental health (F00-F99) and California residency were included. Duplicate records were removed, and multiple visits on the same day were counted once. ED visits with COVID-19-related *ICD-10* codes were excluded due to ambiguity regarding their association with wildfire PM_2.5_ or COVID-19.

**Table 1.** *ICD-10* Codes to Classify Each Disorder

Group	*ICD-10* code
Mental disease	
All mental disease	F00-F99
Mental and behavioral disorders due to psychoactive substance use	F10-F19
Schizophrenia, schizotypal, delusional, and other nonmood psychotic disorders	F20-F29
Other mood affective disorders	F30-F31
Depression	F34-F39
Anxiety	F32, F33

Abbreviation: *ICD-10*, *International Statistical Classification of Diseases and Related Health Problems, Tenth Revision*.

Daily ED visit counts were aggregated by disease groups and date at the level of Zip Code Tabulation Areas (ZCTAs). ZCTAs were selected for their accurate representation of populated areas, consistency in geographic units across datasets, and optimal balance between spatial resolution, data availability, and sufficient sample sizes for statistical analysis. ZCTAs with zero estimated population were excluded.

### Wildfire-Specific PM_2.5_

We used daily, local-level wildfire-specific PM_2.5_ data from a well-validated model developed by Childs et al.^17^ The model employed machine learning techniques, leveraging PM_2.5_ information from the ground station, satellite (smoke plume), and meteorological reanalysis data sources to separate wildfire-specific PM_2.5_ from background PM_2.5_.^17^ Unlike interpolation-based approaches, it avoids understating extreme concentrations caused by missing or sparse monitor data. Moreover, isolating wildfire-specific PM_2.5_ helps reduce the influence of other confounding factors like traffic.

### Covariates

Wildfire data, including dates, locations, acres burned, and causes, were collected from the California Department of Forestry and Fire Protection (CalFire). Climatology variables, such as average humidity and temperature at the ZCTA level, were collected from the Google BigQuery Data Warehouse and ZCTA-level population data from the US Census Bureau. Copollutant data were retrieved from the EPA^18^ and estimated at the ZCTA level using an Inverse Distance Weighted (IDW) interpolation model (eMethods in Supplement 1). The Social Deprivation Index (SDI) was included to account for socioeconomic characteristics.^19,20^

Evacuation order information was collected from CalFire’s official Twitter feed, as no comprehensive dataset was available. From the evacuation announcement, we first identified counties had evacuation orders: Alameda, Colusa, Fresno, Glenn, Humboldt, Lake, Marin, Mendocino, Merced, Napa, Orange, San Benito, San Bernardino, San Diego, San Joaquin, San Mateo, Santa Clara, Santa Cruz, Shasta, Solano, Sonoma, Stanislaus, Tehama, Trinity, and Yolo. We then assumed that ZCTAs within 50 km of wildfires in these counties were potentially affected by evacuation orders, as the US Fire Administration issues emergency alerts to areas within 30 miles of wildfires.

### Statistical Analysis

A population-based epidemiologic analysis was conducted on daily ED visits for mental health conditions and wildfire-specific PM_2.5_ exposure using a distributed lag nonlinear model (DLNM):


*log (E[Y_zct_]) = α + β × WFPM_tl_ + γX_zt_ + ns(time, 3) + ρW × WFPM_zt_ + δ_c_ + log(pop_z_)*

In this model, *Y_zct_* represents the daily ED visit count in ZCTA *z* on date *t*; α is the intercept; *WFPM_tl_* is the cross-basis function of wildfire-specific PM_2.5_ to model nonlinear and lag effects; *ns(time, 3)* represents a natural spline term for time to account for seasonality and temporal trends; *W × WFPM_zt_* represents the spatially lagged PM_2.5_ to capture spatial correlation of exposure, using k-nearest neighbors method (with k equaling 4) and county-level fixed effects *δ_c_* account for time-invariant characteristics. *X_zt_* includes confounding such as daily average temperature, daily average humidity, COVID-19–related ED counts, SDI, baseline mental health ED counts (March to June 2020) to account for pre-fire mental health pattern, season, holidays, day of the week, and proximity to the active fire zone (within 10 km, 50 km, 100 km, or farther) on date *t* in ZCTA *z*. The COVID-19–related ED visit count will adjust its potential influence on patients’ health care-seeking behaviors. The model adjusted for the number of populations at ZCTA by including the natural logarithm of the population.

This model incorporates the immediate (lag 0) and delayed effects (up to 7 days) of wildfire PM_2.5_ on ED visits, considering the persistent wildfire smoke impact and potential treatment delays. A nonlinear exposure-response association was modeled using equal knots of the natural cubic spline of wildfire-specific PM_2.5_. We used a quasi-Poisson generalized linear model to address overdispersion and zero outcomes. To facilitate comparison with previous studies, results are presented as a change in ED counts per 10 μg/m^3^ of wildfire-specific PM_2.5_. Our focus is on understanding the overall acute association of wildfire smoke with human health; therefore, all results, except for immediate outcomes (day 0), are reported as cumulative relative risk (cRR) over lags after exposure.

We conducted demographic-specific stratified analyses to examine potential effect modification by different population groups. Subgroups were defined by age (children [0 to 14 years], youth [15 to 24 years], adult [25 to 64 years], senior [65 years and older]), sex (male, female), race and ethnicity (Hispanic, non-Hispanic Black, non-Hispanic White, other [including American Indian or Alaska Native, Native Hawaiian or other Pacific Islander]), and health insurance types (Medicare, Medicaid, private, other health insurance, self-pay). Race and ethnicity was included as a variable because previous studies on air pollution and wildfire exposure have shown the risk of air pollution and wildfire smoke can be more pronounced in certain racial and ethnic groups.^27,23,24,31^

For sensitivity analyses, we compared areas with and without evacuation orders to address the potential impact of evacuation-related stress due or property loss on health outcomes. Next, we included copollutants to account for their potential impact on associations. Lastly, we restricted analyses to single-visit individuals to ensure the associations were not influenced by recurrent users. Autocorrelation was tested (eMethods in Supplement 1). All analyses were conducted using R version 2024.09.1 + 394 (R Project for Statistical Computing) with the dlnm package.

## Results

There were 86 609 ED visits for mental-related outcomes after applying exclusion criteria from July to December 2020 (median [IQR] age, 38 (27-54) years; 40 272 female [46.5%], 10 657 non-Hispanic Black [12.3%], 35 145 non-Hispanic White [40.6%]) (Table 2). The mean (SD) daily concentration of wildfire-specific PM_2.5_ increased up to 24.9 (34.6) µg/m^3^ during the peak wildfire period in September (maximum, 296 µg/m^3^). Seasonal effects were observed in meteorological factors. A spatial distribution of mean ED visit rates per 100 000 individuals and average wildfire-specific PM_2.5_ can be found in eFigure 2 in Supplement 1.

**Table 2.** Summary Statistics of Counts of ED Visits, Fine Particulate Matter, and Meteorological Variables^a^

Variables	July	August	September	October	November	December
ED visits, No.						
Mental health visits^b^	16 508	16 486	15 586	14 962	12 442	10 625
Individuals	14 794	14 197	13 298	12 386	10 296	8734
Meteorological factors, mean (SD)						
Temperature, °F	71.84 (8.32)	74.86 (8.74)	72.48 (7.90)	67.28 (8.12)	54.58 (8.54)	50.89 (7.47)
Humidity, g/kg	56.79 (20.13)	56.57 (19.47)	54.42 (21.43)	51.03 (21.47)	59.06 (19.56)	58.93 (23.21)
Wind speed, m/s	4.56	4.49	4.12	3.30	2.86	2.33
Ambient PM_2.5_, μg/m^3^	9.24 (5.41)	17.69 (19.72)	33.90 (33.01)	20.99 (20.97)	12.89 (6.91)	13.24 (6.60)
Wildfire-specific PM_2.5_, μg/m^3^	0.44 (1.83)	9.65 (18.4)	24.90 (32.4)	9.33 (14.7)	0.80 (2.68)	0.09 (0.52)
Population-weighted monthly PM_2.5_ in California, μg/m^3^	0.10 (0.19)	3.95 (4.87)	10.20 (9.95)	4.39 (4.17)	0.39 (0.82)	0.06 (0.11)
Time wildfire specific PM_2.5_ >15 ug/m^3^, d^c^	0.1	6.0	14.1	5.21	0.27	0
Total active wildfires, No.	16	41	40	26	23	10

Abbreviations: ED, emergency department; PM_2.5_, fine inhalable particles that are 2.5 µm and smaller.

^a^ Monthly number of fires and meteorological conditions during the study period, July 1 to December 31, 2020. Fine particulate matter defined as PM_2.5_, or inhalable particles that are 2.5 µm and smaller. Both ambient and wildfire-specific PM_2.5_ are 3-day moving averages.

^b^ Total number of ED visits for mental health visits using primary diagnosis code from California residents.

^c^ The number of days exceeding 15 μg/m^3^ was based on the World Health Organization (WHO) updated guidelines which state that 24-hour average exposures should not exceed 15 ug/m3 more than 3-4 days per year. Source: WHO global air quality guidelines.^30^

**Figure 1.**
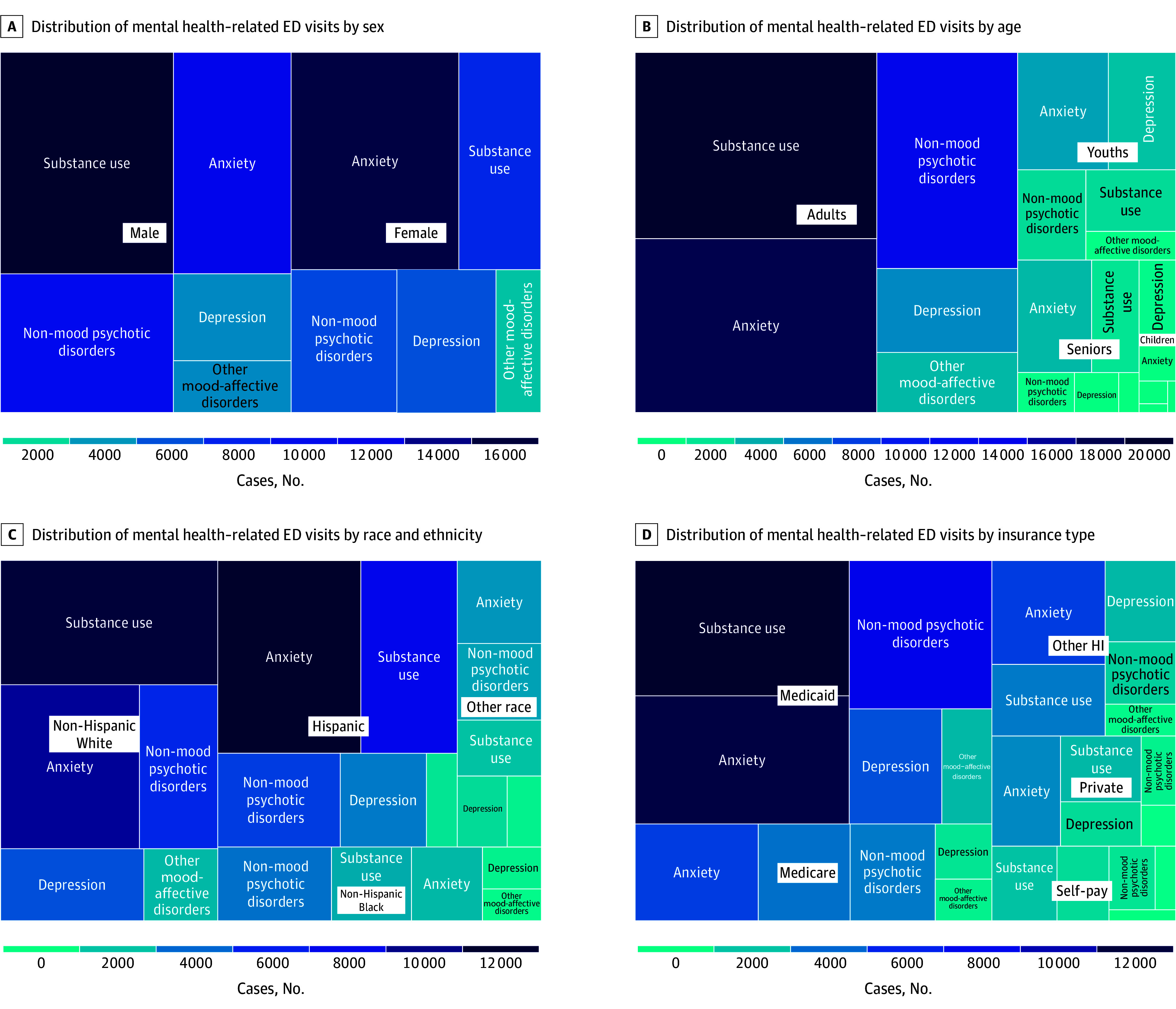
Distribution of Emergency Department (ED) Visits for Mental Health–Related Causes by Demographic Characteristics HI indicates health insurance.

Figure 1 visualizes the distribution of mental health–related ED visits by demographic characteristics. Substance use disorder was the leading cause for male individuals (16 360 visits [35.3%]), while anxiety was most common among female individuals (15 573 visits [38.7%]) (Figure 1A). Substance use disorder was the primary cause among non-Hispanic White (11 510 visits [32.8%]), anxiety for Hispanic (11 775 visits [39.2%]), and nonmood psychotic disorders for non-Hispanic Black individuals (3581 visits [33.6%]) (Figure 1B). Substance use was the most common cause for adults (19 221 visits [32.0%]), while anxiety was more prevalent among youth (4552 visits [31.6%]) and seniors (3557 visits [37.3%]), and depression led for children (1235 visits [47.0%]) (Figure 1C). Anxiety was the most common across all insurance types (Medicare, 5090 visits [30.9%]; other health insurance, 5020 visits [35.4%]; private, 3228 visits [36.3%]), except for Medicaid or self-pay, where substance use was more frequent (Medicaid, 12 371 visits [30.1%]; self-pay, 1656 visits [27.8%]) (Figure 1D).

**Figure 2.**
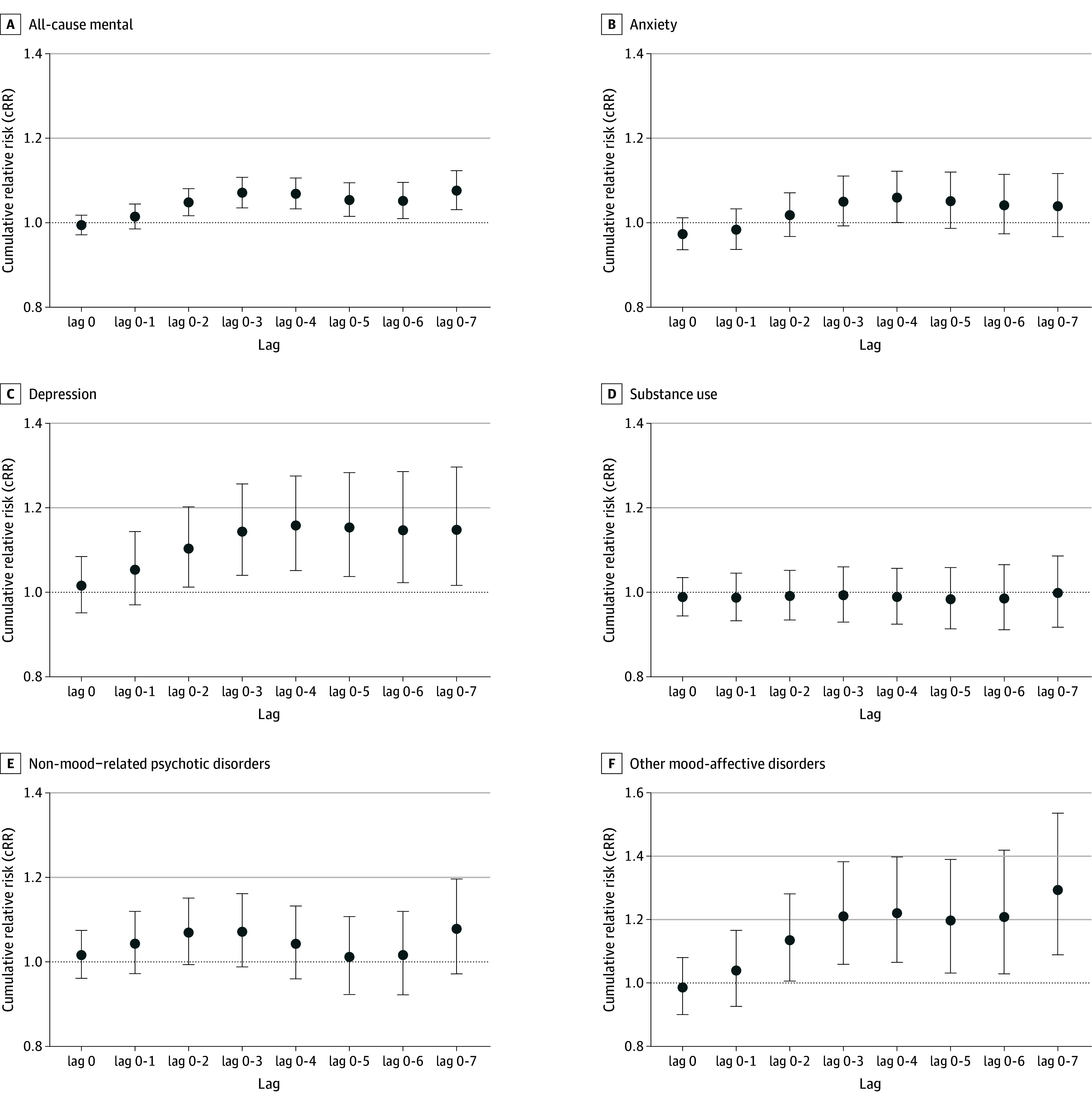
Cumulative Relative Risk (cRR) for Mental Health–Related Emergency Department Visits Following Wildfire-Related PM_2.5_ Increase Relative risk in exposure day (lag 0) and cumulative relative risk (delayed effects) and 95% CIs per 10-μg/m3 increase in wildfire specific PM_2.5_ by outcome for all population (overall).

Figure 2 presents the cRR of wildfire-specific PM_2.5_ on ED visits for mental health conditions across different cumulative lags. A 10-μg/m^3^ increase in wildfire-specific PM_2.5_ was significantly associated with increased ED visits for all-cause mental health conditions (cRR, 1.17; 95% CI, 1.03-1.12), depression (cRR, 1.15; 95% CI, 1.02-1.30), and other mood-affective disorders (cRR, 1.29; 95% CI, 1.00-1.45) up to 7 days postexposure. Additionally, ED visits for anxiety showed a delayed association with ED visits, with an increase observed 3 days after exposure.

**Figure 3.**
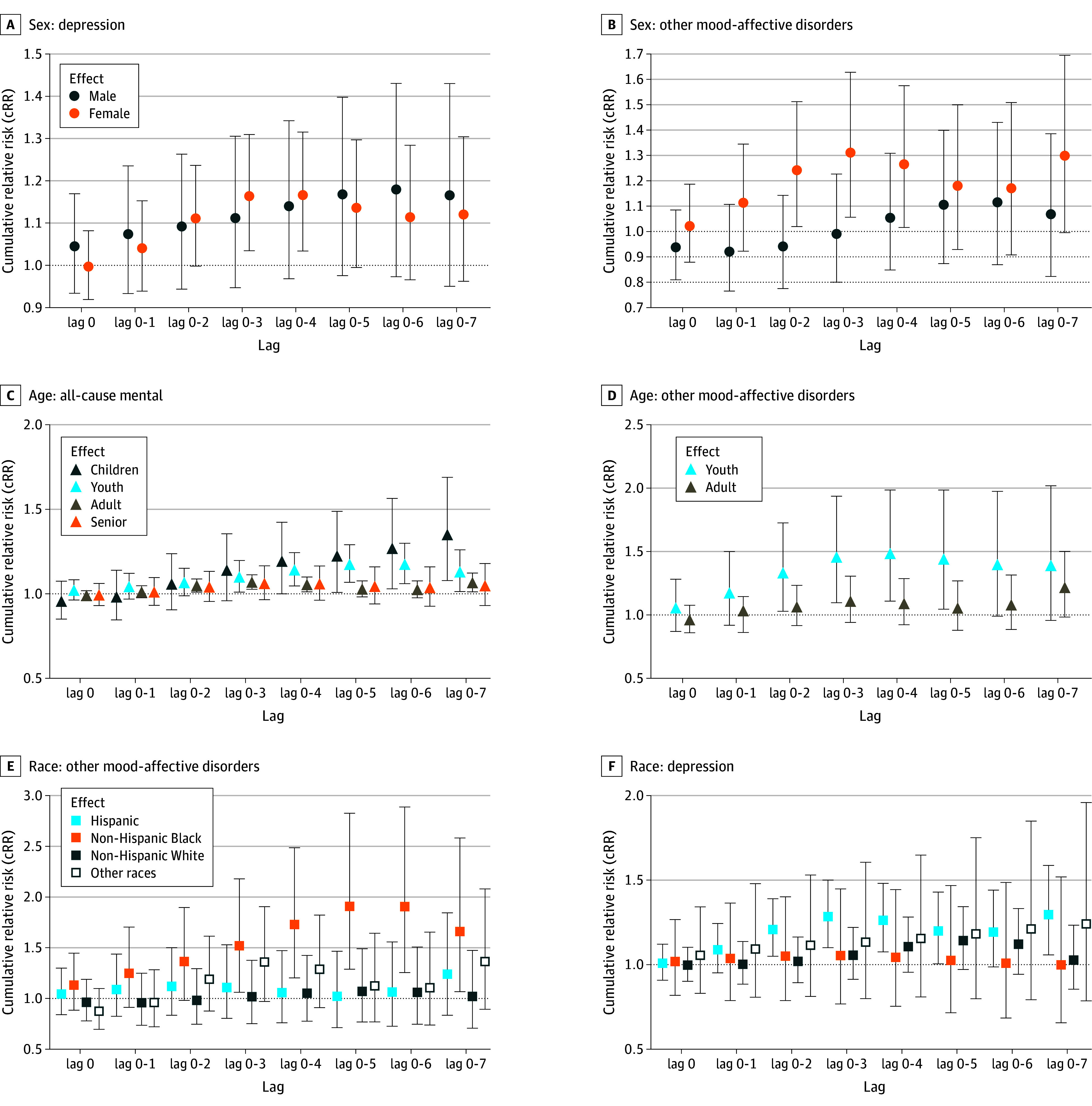
Effect Modifiers of the Association Between Wildfire-Specific PM_2.5_ and ED Visits Across Multiple Lag Periods Relative risk in exposure day (lag 0) and cumulative relative risk (delayed effects, lags up to 7 days) and 95% CIs per 10-μg/m3 increase in wildfire-specific PM_2.5_ on specific mental health conditions.

Women had an increased risk of ED visits for depression, with a significant association observed at lag 0 to 4 days (cRR, 1.17; 95% CI, 1.03-1.32) (Figure 3A). Women were also more likely to visit EDs for other mood-affective disorders within 3 days following exposure (cRR, 1.34; 95% CI, 1.11-1.62) (Figure 3B). Exposure to wildfire-specific PM_2.5_ led to higher risks of all-cause ED visits up to 7 days (cRR, 1.44; 95% CI, 1.16-1.79) and anxiety visits up to 6 days (cRR, 1.57; 95% CI, 1.08-2.29) (Figure 3C). Youth showed increased ED visits for other mood-affective disorders up to 4 days (cRR, 1.46; 95% CI, 1.08-1.98) (Figure 3D). Non-Hispanic Black individuals had a higher risk of ED visits for other mood-affective disorders within lag 0 to 5 days (cRR, 2.35; 95% CI, 1.56-3.53) (Figure 3E). Hispanic individuals had an increased risk of ED visits for depression (cRR, 1.30; 95% CI, 1.06-1.59) up to 7 days postexposure (Figure 3F). The association of wildfire-specific PM_2.5_ exposure with mental health ED visits across sociodemographic characteristics are explored in eFigure 3 in Supplement 1. Stratified analysis by insurance type showed that only Medicaid holders experienced significant increases in the risk of ED visits for all-cause mental health conditions and depression, particularly at a later lag (eFigure 3D in Supplement 1).

In our sensitivity analyses, we assessed whether the association between wildfire smoke and outcomes was modified by areas potentially affected by evacuation orders (eFigure 3E in Supplement 1). The results showed no distinct differences between areas with and without evacuation orders, although wildfire-specific PM_2.5_ was linked to increased other mood-affective disorders shortly after the wildfire (lag 0 to 3 days) in both areas, with decreases observed after 3 days for affected areas. The copollutants adjusted model showed consistent results with the main analysis (eFigure 4 in Supplement 1). Finally, we restricted the analysis to individuals with a single visit to ensure that frequent ED users were not driving the association. It yielded similar findings consistent with the main analysis, indicating robustness (eFigure 5 in Supplement 1).

## Discussion

Our study investigated the association between short-term wildfire-specific PM_2.5_ exposure during the 2020 California wildfire season and mental health. We observed positive associations between wildfire-specific PM_2.5_ and all-cause mental health conditions, depression, and other mood-affected disorders, after adjusting for potential confounders. Effect modification was observed in certain groups, including female individuals, Black and Hispanic individuals, and Medicaid holders.

Our findings align with previous studies on AAP and mental health.^21,22^ Short-term AAP studies reported a relative risk (RR) of 1.01 (95% CI, 1.00-1.02) for emergency admissions for ED visits per 10-μg/m^3^ increase in 2-day average PM_2.5_.^21^ A UK biobank study found the odds of mental disorders and major depression increased by 2.31 and 2.26 times, respectively, per 10-μg/m^3^ increase in PM_2.5_.^13^ Similarly, we found an 8% increase (percentage changes as calculated by *[1  cRR] × 100*) in all-cause mental health ED visits, a 15% increase in depression, and a 29% increase in other mood-affective disorders per 10 μg/m^3^ PM_2.5_ over 7 days, with larger effect sizes than AAP studies. A 2024 study on wildfire smoke and ED visits for anxiety disorders found a 6.3% increase.^9^ We also observed a slightly greater 6% increase in anxiety-related visits up to 4 days postexposure during the 2020 wildfire season.

For risk differences, we identified that female individuals experienced a greater effect of wildfire smoke exposure compared with their male counterparts, consistent with previous studies.^9,23^A review of respiratory sex differences found female individuals may be more susceptible to specific respiratory conditions, including COPD and asthma. One study^24^ reported a 10.4% increased risk of respiratory admissions for women on smoke-wave days compared to men. Also, Zhu et al^9^ reported an increased risk of anxiety-related ED visits among girls and women due to wildfire smoke exposure, similar to our findings. Previous research suggests that AAP, particularly PM_2.5_, may affect hormone levels and act as an endocrine disruptor, influencing stress responses and mental health issues.^25^ These effects, combined with differences in neural circuitry and mechanisms by sex, could explain why female individuals may be more vulnerable to anxiety and depression from wildfire exposure.^26^

Our findings suggest that children and youth may be more susceptible to wildfire-specific PM_2.5_ exposure, aligning with epidemiological studies on AAP and wildfire smoke.^24,25^ Children and youths showed the risk of ED visits for mental health conditions was 35.0% and 17.4% higher per 10-ug/m3 increase in wildfire-specific PM_2.5_. Increased ED visits for depression and other mood-affective disorders were also observed among youth. Previous research on AAP has identified associations between adolescent psychotic experiences and AAP exposure.^11,25^ Childhood and adolescence, critical periods for brain development, may be particularly vulnerable to the toxicity of wildfire smoke, potentially increasing the risk of mental health disorders.^26,27^

Race-stratified analysis suggested that individuals from minoritized groups may be more vulnerable to wildfire smoke, consistent with previous findings on racial disparities in air pollution.^27,28^ Hispanic individuals are more likely to experience depressive episodes than other racial groups.^29^ This vulnerability may be exacerbated by wildfire smoke, potentially increasing the risk of ED visits for depression. Additionally, non-Hispanic Black individuals showed a heightened risk of ED visits for other mood-affective disorders. These findings highlight the importance of addressing racial disparities in air pollution and mental health outcomes. Overall, our subgroup analysis emphasizes the importance of strategies to ensure equal protection from wildfire exposure across demographic groups.

Our study focused on mental health outcomes related to smoke exposure rather than traumatic events like evacuations or physical injuries. While stress from evacuations and property loss could contribute to increased ED visits, sensitivity analyses showed similar trends in areas affected by evacuation orders and those not. ED visits related to other mood-affective disorders temporarily increased, possibly due to stress induced by evacuation orders, while areas without evacuation orders showed a small but consistent increased risk that might be due to prolonged wildfire smoke exposure. However, further studies with more detailed evacuation data are needed.

### Strengths and Limitations

A major strength of our study, compared with prior wildfire studies, is its statewide analysis of HCAI data, incorporating time-varying spatial exposure to wildfire-specific PM_2.5_ during the 2020 wildfire seasons. Unlike other wildfire studies limited to specific areas, our approach provides a more representative understanding of exposure across California. Using wildfire-specific PM_2.5_ data offers a more accurate estimate of the health impact compared with general PM_2.5_ data. Adopting the DLNM allowed us to understand the delayed effect of PM_2.5_ on ED visits, identifying the critical periods with the most pronounced effect sizes. Furthermore, our subgroup analysis, incorporating sociodemographic factors, helped identify populations vulnerable to wildfire smoke.

Our study has several limitations. First, ED visit volumes may be underestimated due to data collection during the COVID-19 pandemic. However, we found a positive link between wildfire-specific PM_2.5_ and mental health ED visits even after we controlled for COVID-19 cases and baseline visits as a potential confounder. Patients presenting with both mental health issues and COVID-19 were excluded from this analysis due to unclear causality, which may introduce bias, particularly given the potential for COVID-19 to disproportionately affect individuals based on race and socioeconomic status. Although we attempted to separate the impacts of evacuation orders and wildfire smoke exposure, limited data availability may have prevented us from fully distinguishing between the two. Additionally, using retrospective claims databases for hospital billings and diagnostic and/or procedure codes may introduce inaccuracies, although this should not compromise internal validity unless coding errors were linked to exposure. Individual-level exposure, lifestyle factors, or behavior changes were unavailable. Lastly, we did not adjust for multiple comparisons, so *P* values and confidence intervals should be interpreted accordingly.

## Conclusions

In this cross-sectional study of wildfire smoke exposure, we found a positive association between wildfire smoke and mental health–related ED visits. We identified this association may be modified by sex, race and ethnicity, age, or insurance type. These results highlight the importance of health care professionals and systems anticipating a possible increase in demand for mental health services in ED during wildfire events.

## Notes

Supplement 1.
eMethods.
**eTable.** Top 10 Wildfires in California During the Study Period January-December 2020 **eFigure 1.** Wildfire Perimeters With Areas Larger Than 1000 Acres During July 1-December 31, 2020 **eFigure 2.** Mean Values of Wildfire-Specific PM_2.5_ and Rate of Emergency Department (ED) Visits During the Study Period (July-December 2020) by ZCTA **eFigure 3.** Effect of Wildfire-Related PM_2.5_ on ED Visits by Multiple Lag Periods, Age, Gender, Race/Ethnicity, and Health Insurance Type **eFigure 4.** Sensitivity Analysis of the Effect of Wildfire-Related PM_2.5_ on ED Visits Adjusted for Co-Pollutants **eFigure 5.** Sensitivity Analysis Comparing the Effect of Wildfire-Related PM_2.5_ on ED Visits Between Main Results and Single-Visit Cases

Supplement 2.
Data Sharing Statement
