# Error in Funding/Support

**DOI:** 10.1001/jamanetworkopen.2025.22954

**Published:** 2025-06-27

In the Original Investigation titled “Fine Particulate Matter From 2020 California Wildfires and Mental Health–Related Emergency Department Visits,”^1^ published April 4, 2025, grant R01ES032253 from the National Institutes of Environmental Health Sciences was omitted from the Funding/Support section. This article has been corrected.^1^
