# Supplementary material for: Fine Particulate Matter From 2020 California Wildfires and Mental Health–Related Emergency Department Visits
Source: JAMA Netw Open. 2025 Apr 4;8(4):e253326. doi: 10.1001/jamanetworkopen.2025.3326 (PMC11971671; doi:10.1001/jamanetworkopen.2025.3326)
Supplement: Supplement 1. — eMethods. eTable. Top 10 Wildfires in California During the Study Period January-December 2020 eFigure 1. Wildfire Perimeters With Areas Larger Than 1000 Acres During July 1-December 31, 2020 eFigure 2. Mean Values of Wildfire-Specific PM2.5 and Rate of Emergency Department (ED) Visits During the Study Period (July-December 2020) by ZCTA eFigure 3. Effect of Wildfire-Related PM2.5 on ED Visits by Multiple Lag Periods, Age, Gender, Race/Ethnicity, and Health Insurance Type eFigure 4. Sensitivity Analysis of the Effect of Wildfire-Related PM2.5 on ED Visits Adjusted for Co-Pollutants eFigure 5. Sensitivity Analysis Comparing the Effect of Wildfire-Related PM2.5 on ED Visits Between Main Results and Single-Visit Cases [file jamanetwopen-e253326-s001.pdf]

Supplemental Online Content

Jung YS, Johnson MM, Burke M, et al. Fine Particulate Matter from 2020 California wildfires and mental health-related emergency department visits. *JAMA Netw Open.* 2025;8(4):e253326. doi:10.1001/jamanetworkopen.2025.3326

eMethods.

eTable. Top 10 Wildfires in California During the Study Period January-December 2020

eFigure 1. Wildfire Perimeters With Areas Larger Than 1000 Acres During July 1-December 31, 2020

eFigure 2. Mean Values of Wildfire-Specific $PM_{2.5}$ and Rate of Emergency Department (ED) Visits During the Study Period (July-December 2020) by ZCTA

eFigure 3. Effect of Wildfire-Related $PM_{2.5}$ on ED Visits by Multiple Lag Periods, Age, Gender, Race/Ethnicity, and Health Insurance Type

eFigure 4. Sensitivity Analysis of the Effect of Wildfire-Related $PM_{2.5}$ on ED Visits Adjusted for Co-Pollutants

eFigure 5. Sensitivity Analysis Comparing the Effect of Wildfire-Related $PM_{2.5}$ on ED Visits Between Main Results and Single-Visit Cases

This supplemental material has been provided by the authors to give readers additional information about their work.

© 2025 Jung YS et al. *JAMA Network Open*.

Supplemental Material, Section A: Air pollution exposure estimation and analysis

For the sensitivity analysis, we calculated daily co-pollutant levels, including ozone (O3), nitrogen dioxide (NO2), and carbon monoxide (CO), to address potential confounding. For validation, we estimated daily ground-level $PM_{2.5}$ exposure using daily summary PM2.5 FRM/FEM Mass (88101) data. We downloaded the daily average air quality data sets from the US Environmental Protection Agency's (EPA) Air Quality System and applied inverse distance-squared weighting (IDW) for interpolation. Up to four closest monitoring stations within 50km of the patient's home zip code were considered, excluding near-road sites, as these only represent conditions along major roadways. We calculated the distance between a residential zip code and stations and calculated the weighted concentration using air pollutant measurement from the closest station. If the nearest monitor is within 0.25km, we only use data from that monitor.

Supplemental Material, Section B: Autocorrelation Check

To assess the robustness of our quasi-Poisson regression analysis, we performed several diagnostic checks on our model. First, we examined the goodness-of-fit by evaluating the deviance and Pearson residuals. These diagnostics help to determine whether the model adequately captures the relationship between the exposure (wildfire-specific PM2.5) and the mental health outcomes. Deviance residuals and Pearson residuals were inspected for any large deviations, which could indicate misspecification or poor fit, and our results suggested that the model fit the data well.

Next, we assessed the presence of overdispersion, which occurs when the variance of the outcome exceeds the mean. We checked the ratio of the residual deviance to the degrees of freedom as a measure of overdispersion. For all models, this ratio was below 1, suggesting there was no evidence of overdispersion in our data, and thus, our model assumptions were appropriate.

Finally, we evaluated autocorrelation in the residuals, which could indicate that our model did not fully account for time-dependent structures or spatial correlation. We used autocorrelation function (ACF) plots to assess the residuals for autocorrelation. For the main analysis, all of our ACF plots showed values near zero, indicating minimal autocorrelation, which suggests that the model adequately accounted for temporal and spatial dependencies in the data.

© 2025 Jung YS et al. *JAMA Network Open*.

Supplemental Material, eTable 1. Top 10 wildfires in California during the study period January – December 2020

Fire name	Start date	End date	Reported acres
August Complex	August 15, 2020	November 10, 2020	1,032,699*
SCU Complex	August 15, 2020	September 10, 2020	396,824*
Creek	September 3, 2020	December 23, 2020	379,895
North Complex	August 16, 2020	December 2, 2020	318,776
Hennessey	August 16, 2020	September 15, 2020	305,651
Castle	August 18, 2020	January 3, 2021	174,178
Slater	September 7, 2020	December 11, 2020	157,270
Red Salmon Complex	July 26, 2020	November 18, 2020	142,386
Dolan	August 17, 2020	December 30, 2020	124,924
Bobcat	September 5, 2020	October 18, 2020	115,796

*If reported acres were unavailable, we used GIS Acres
source: Fire and Resource Assessment Program of the California Department of Forestry and Fire Protection (CalFire)

© 2025 Jung YS et al. *JAMA Network Open*.

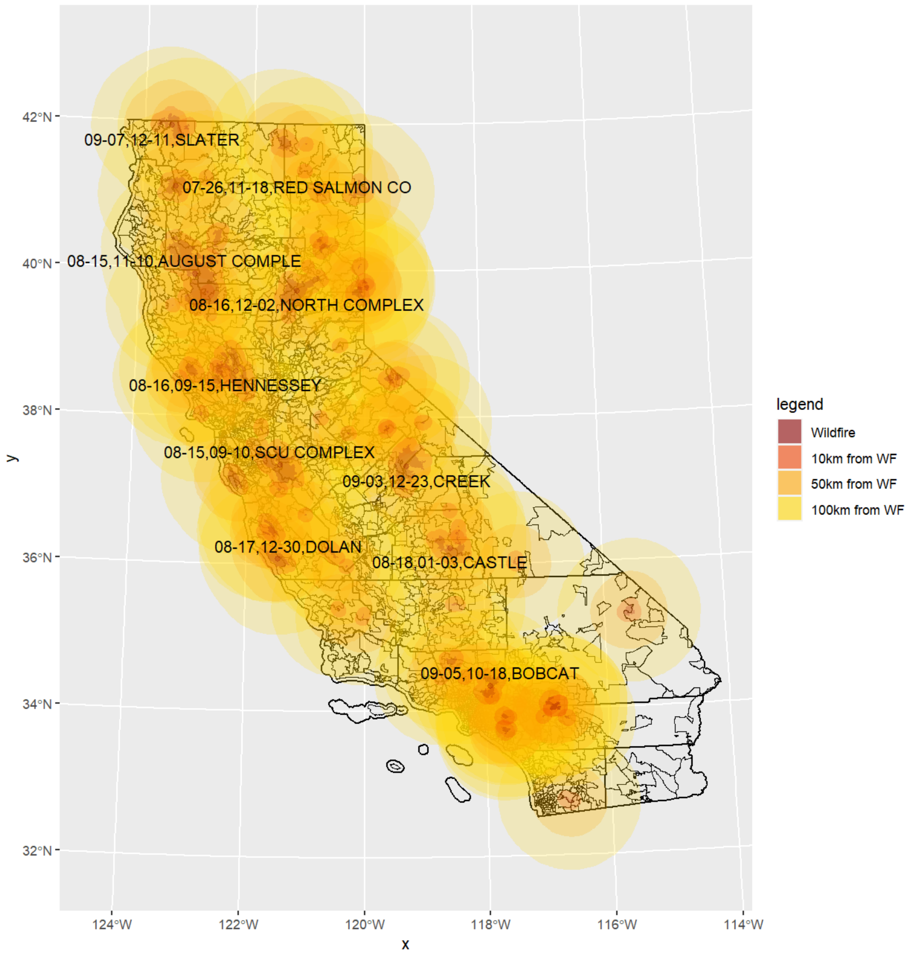

Supplemental Material, eFigure 1. Wildfire perimeters with areas larger than 1,000 acres during July 1st – December 31st, 2020

Note: The 2020 California wildfires burned continuously throughout the second half of the year and occurred all over California, and the size and intensity of wildfires were large. A total of 98 wildfires burned more than 1,000 acres, active for an average of 48 days (min:2, max=140, median=35). Using wildfire perimeters from California in 2020, we mapped the wildfire events larger than 1,000 acres that occurred from July through December 2020. The red-colored areas represent the actual wildfire perimeters during the study period. Since wildfire smoke can travel long distances, we plotted buffers around the polygon of each wildfire perimeter using different distances 10km, 50km, and 100km. With buffers, it was possible to see that most of California's areas were affected by wildfires in 2020. We have also labeled the top 10 wildfires during 2020.

© 2025 Jung YS et al. *JAMA Network Open*.

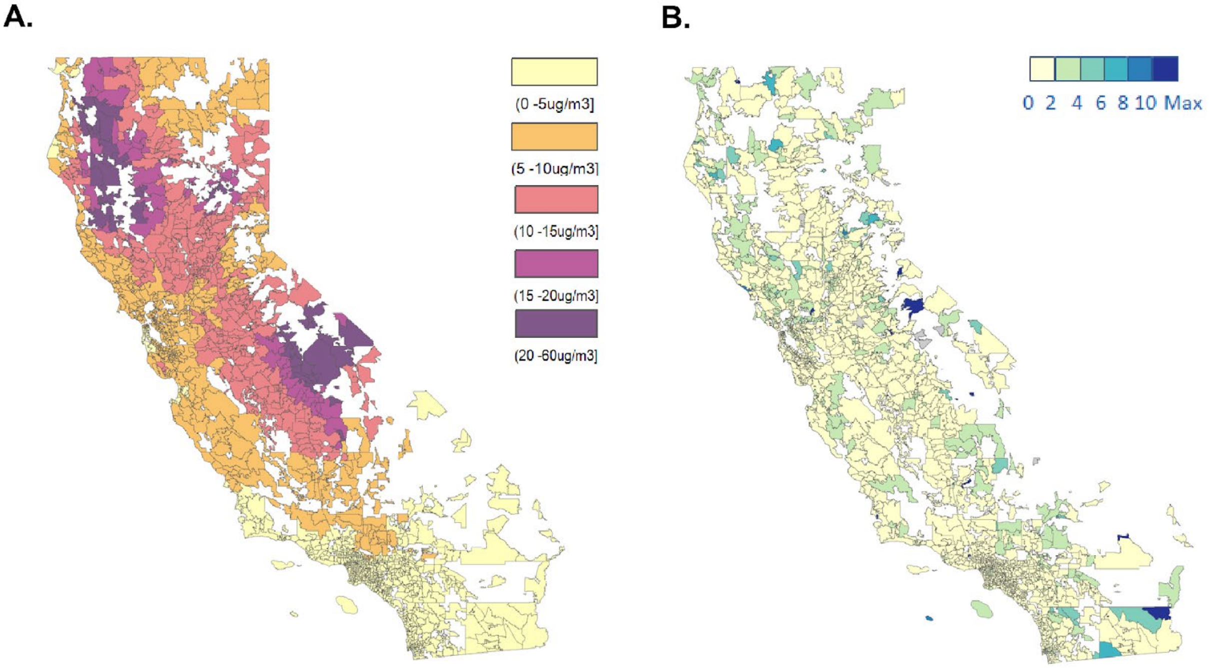

Supplemental Material, eFigure 2. Mean values of wildfire-specific $PM_{2.5}$ and rate of emergency department (ED) visits during the study period (July – December 2020) by ZCTA

Note: (A) Average wildfire-specific PM2.5 concentrations at available ZCTA and (B) average rate of all mental health-related ED visits (per 100,000 individuals) per ZCTA during the study period. (A) The distribution of mean wildfire-specific PM2.5 concentrations was similar to the large-size wildfire distribution as in Figure 1. (B) ZCTAs without ED visits are colored gray. All the rates greater than 10 per 100,0000 were dispersed.

© 2025 Jung YS et al. *JAMA Network Open*.

Supplemental Material, eFigure 3 A-D. Effect of wildfire-related PM2.5 on ED visits by multiple lag periods, age, gender, race/ethnicity, and health insurance type

eFigure 3A. Stratified Analysis by Sex

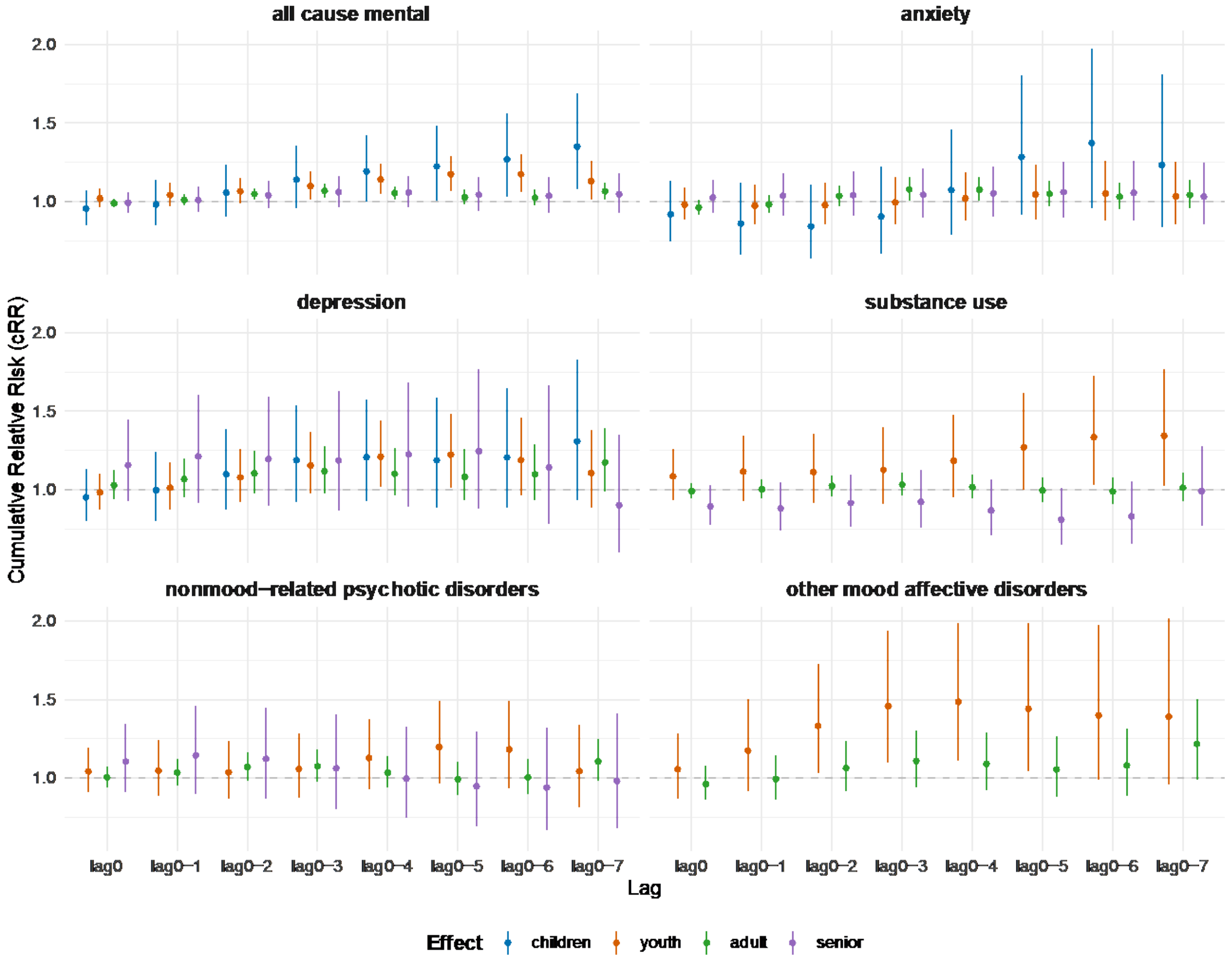

© 2025 Jung YS et al. *JAMA Network Open*.

eFigure 3B. Stratified Analysis by Age

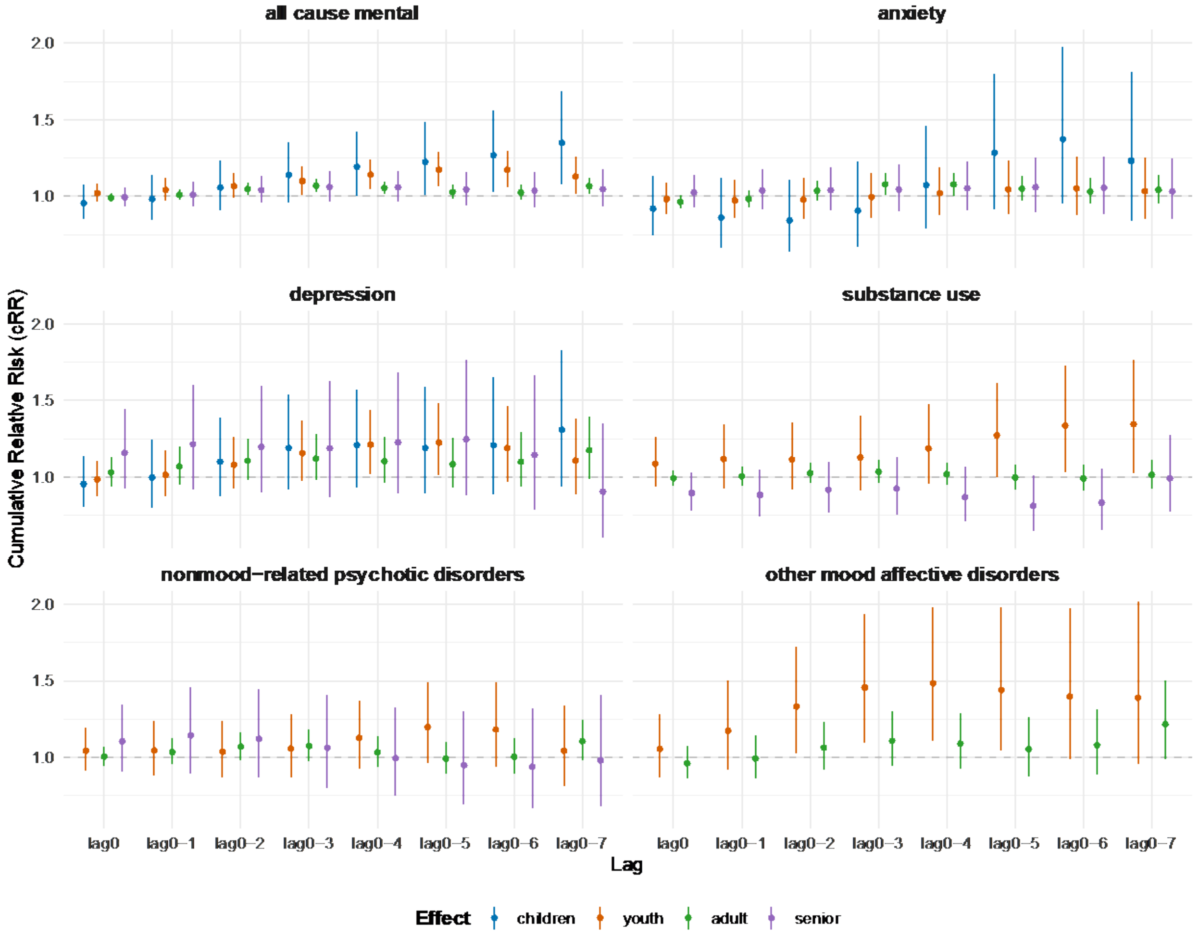

© 2025 Jung YS et al. *JAMA Network Open*.

eFigure 3C. Stratified Analysis by Races

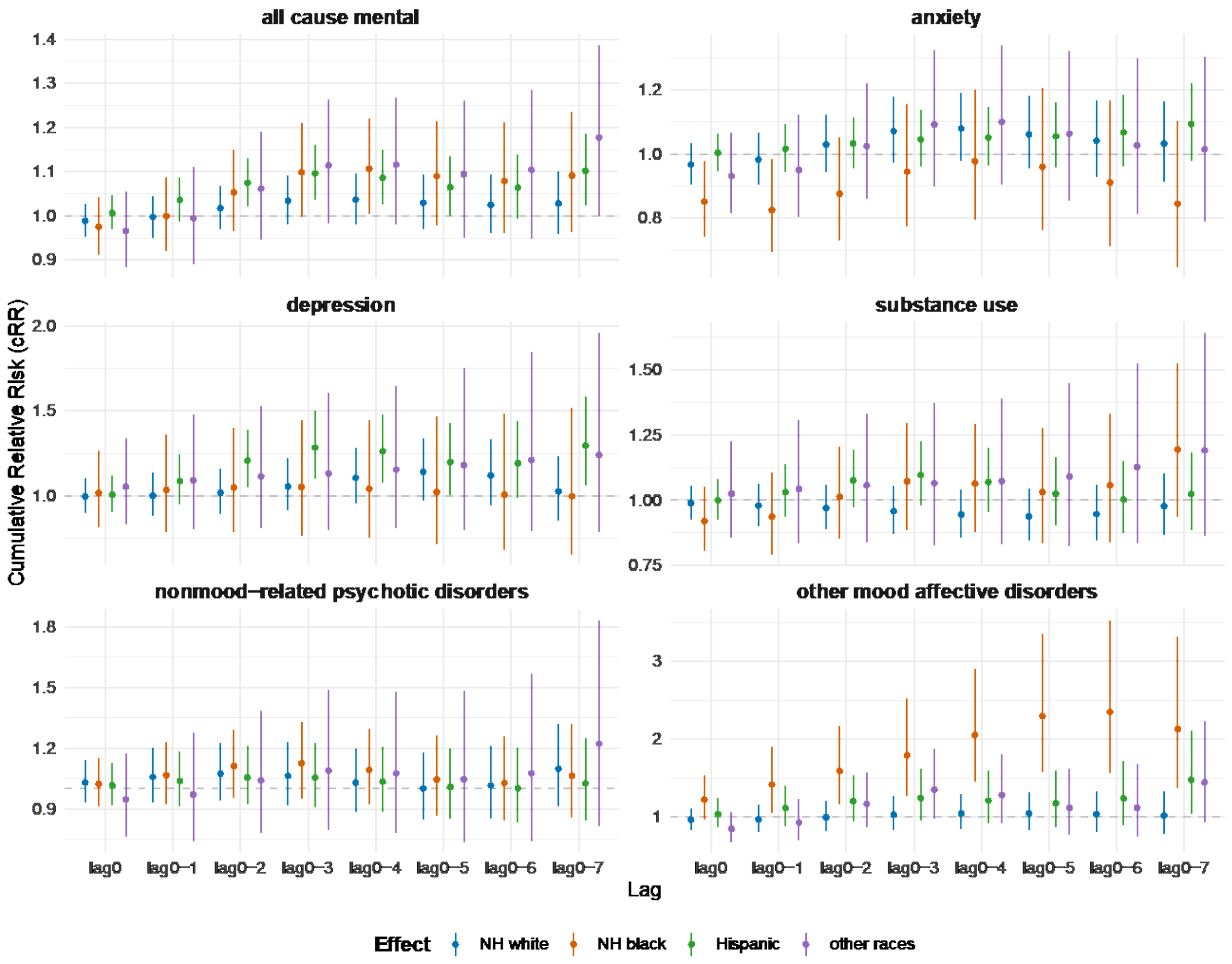

© 2025 Jung YS et al. *JAMA Network Open*.

eFigure 3D. Stratified Analysis by Health Insurance

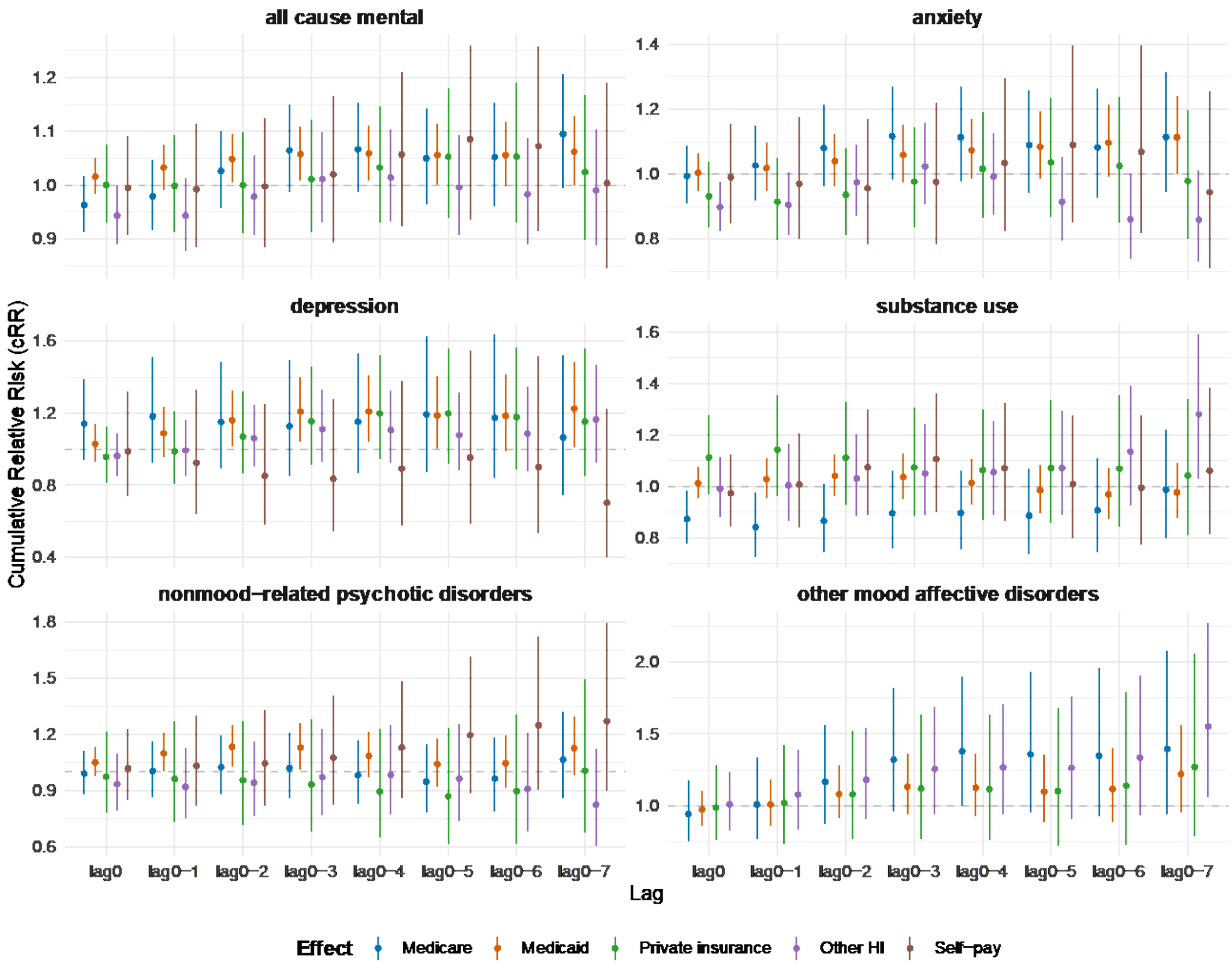

© 2025 Jung YS et al. *JAMA Network Open*.

eFigure 3E. Stratified Analysis by Evacuation Status

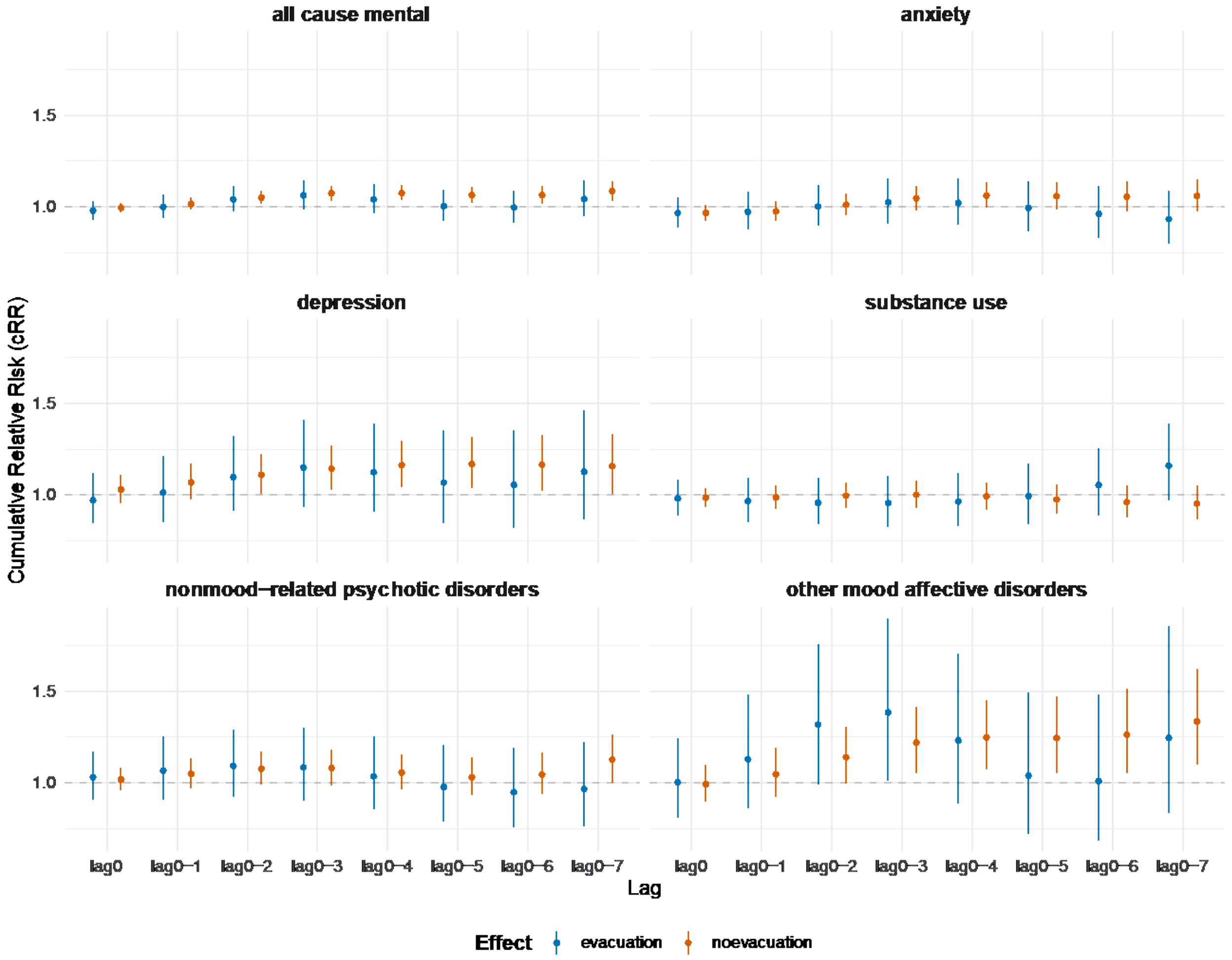

Note: Relative risk in exposure day (lag 0) and cumulative relative risk (delayed effects: lags 0-1,0-2,0-3,0-4, 0-5,0-6, and 0-7) and 95 % confidence intervals per 10 µg/m3 increase in wildfire-specific $PM_{2.5}$ on all-caused ED visits for mental health conditions by (A) age group (children, youth, adult, senior), (B) by sex (female and male), (C) by race/ethnicity (self-reported as Hispanic, non-Hispanic white, non-Hispanic black, and other races), (D) by payer information (Medicare, Medicaid, private, self-insured, and other HI), and (E) by home resident zip code in an area with and without evacuation orders. Only outcomes with sufficient counts (greater than n=500) are given.

© 2025 Jung YS et al. *JAMA Network Open*.

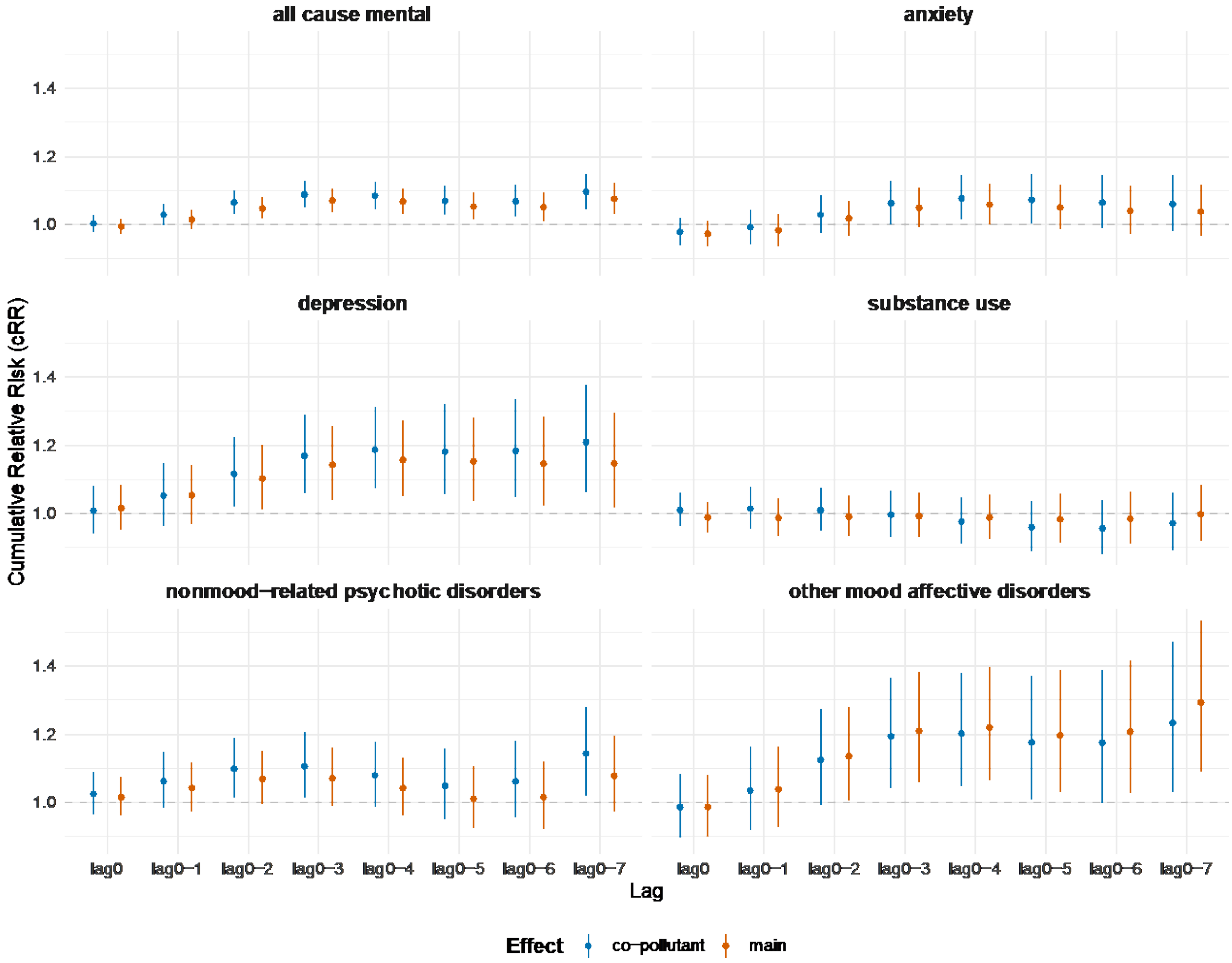

Supplemental Material, eFigure 4: Sensitivity Analysis of the Effect of Wildfire-Related PM2.5 on ED Visits Adjusted for Co-Pollutants

Note: Relative risk for exposure on the same day (lag 0) and cumulative relative risk (delayed effects: lags 0-1, 0-2, 0-3, 0-4, 0-5, 0-6, and 0-7) with 95% confidence intervals per 10 μg/m³ increase in wildfire-specific PM2.5 on all-cause ED visits for mental health conditions, after adjusting for daily average co-pollutants levels (CO, NO2, and Ozone) from EPA. Blue indicates the model adjusted for co-pollutants, and orange represents our main analysis.

© 2025 Jung YS et al. *JAMA Network Open*.

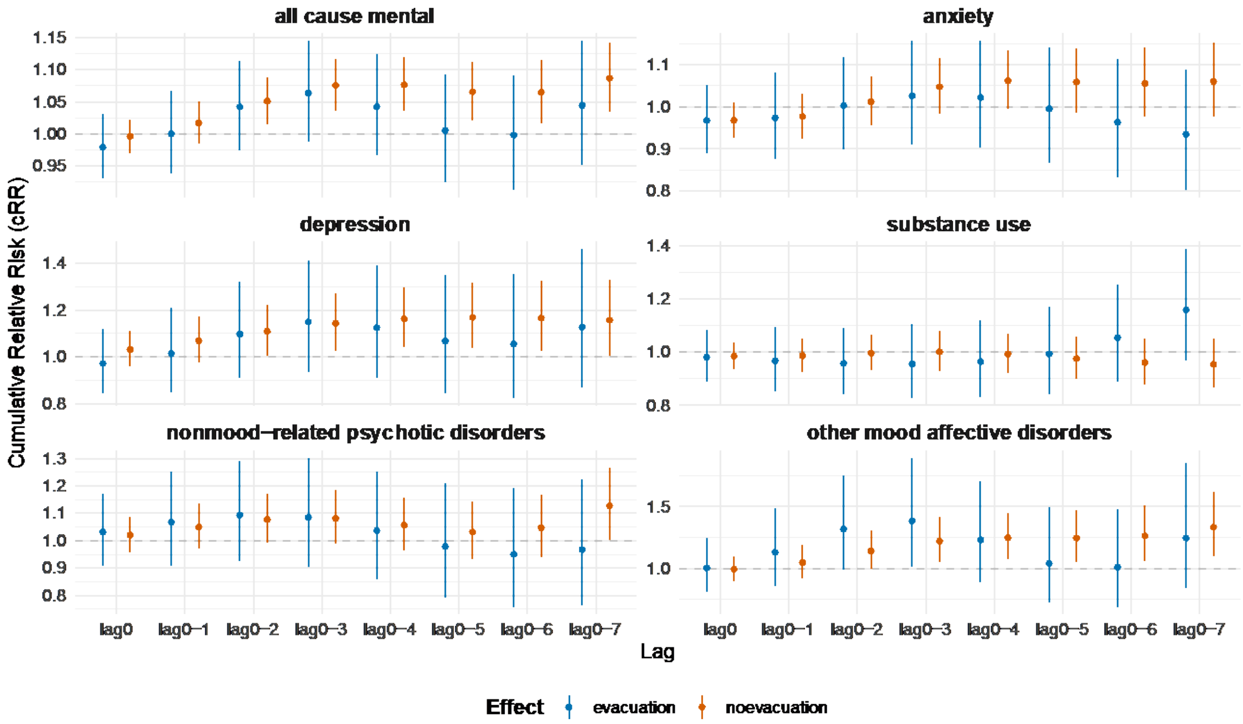

Supplemental Material, eFigure 5: Sensitivity Analysis Comparing the Effect of Wildfire-Related PM2.5 on ED Visits Between Main Results and Single-Visit Cases

Note: Relative risk for exposure on the same day (lag 0) and cumulative relative risk (delayed effects: lags 0-1, 0-2, 0-3, 0-4, 0-5, 0-6, and 0-7) with 95% confidence intervals per 10 µg/m³ increase in wildfire-specific PM2.5 on all-cause ED visits for mental health conditions, after adjusting for daily average co-pollutants levels (CO, NO2, and Ozone) from EPA. Blue bars represent models restricted to individuals with only one ED visit during the study period, while orange bars represent the main analysis including all ED visits.

© 2025 Jung YS et al. *JAMA Network Open*.
