# Supplementary material for: Fine Particulate Matter From 2020 California Wildfires and Mental Health–Related Emergency Department Visits
Source: JAMA Netw Open. 2025 Apr 4;8(4):e253326. doi: 10.1001/jamanetworkopen.2025.3326 (PMC11971671; doi:10.1001/jamanetworkopen.2025.3326)
Supplement: Supplement 2. — Data Sharing Statement [file jamanetwopen-e253326-s002.pdf]

Data Sharing Statement

Jung. Fine Particulate Matter from 2020 California Wildfires and Mental Health–Related Emergency Department Visits. *JAMA Netw Open*. Published April 04, 2025.
doi:10.1001/jamanetworkopen.2025.3326

Data
Data available: No

Additional Information
Explanation for why data not available: Unfortunately, the HCAI data used for this study cannot be shared with others. We are required to discard the data once the project is completed. However, the other data sets are already publicly available.
